# Aortopulmonary collaterals in single ventricle patients

**DOI:** 10.1186/1532-429X-11-S1-P262

**Published:** 2009-01-28

**Authors:** Lars Grosse-Wortmann, Shi-Joon Yoo

**Affiliations:** The Hospital for Sick Children, Toronto, ON Canada

**Keywords:** Pulmonary Vein, Superior Vena Cava, Contrast Magnetic Resonance Imaging, Single Ventricle, Fontan Operation

## Introduction

Aortopulmonary collaterals (APCs) are frequent prior to and following the Fontan operation. APCs have been reported to be associated with prolonged pleural effusions and increased mortality after the Fontan operation. The degree of collateral formation is difficult to measure. A semiquantitative assessment via fluoroscopic angiography is dependent on the amount and location of contrast medium injected.

## Purpose

To i) quantify APC flow in patients after bidirectional cavopulmonary connections (BCPC) and Fontan operations, using phase contrast magnetic resonance imaging (MRI), ii) assess the accuracy of flow measurements in these patients, iii) identify risk factors for the development of APCs.

## Methods

24 MRI studies on 16 patients were analyzed retrospectively. The imaging protocol included phase contrast flow velocity mapping of the superior vena cava (SVC), right and left pulmonary arteries, all individual pulmonary veins, ascending (AAO) and descending aorta (DAO) and in the azygos/hemiazygos venous system. The patients were stratified into 2 groups: A) before and B) after their Fontan completion. APC blood flow was calculated by subtracting the volume of blood flow in the pulmonary arteries from that in the pulmonary veins.

## Results

Qp/Qs was 0.93 ± 0.26 in group A and 1.27 ± 0.16 in group B. In groups A and B 53.3% ± 16.8 and 27.5% ± 15.1, respectively, of total pulmonary blood flow was provided by APCs. The mean inaccuracies, comparing DAO and pulmonary venous flows to that in the AAO in group A and the sum of SVC, DAO and collateral flows to that in the AAO in group B, were 0.26 ± 0.48 l/min/m^2^ (corresponding to 7.9 ± 14.5% of AAO flow) and 0.18 ± 0.43 l/min/m^2^ (corresponding to 7.1 ± 13.6% of AAO flow), respectively. For both groups combined, the degree of left to right shunting showed a positive correlation with the arterial oxygen saturations (r = 0.73, p < 0.0001). The percentage of flow via APCs within the total pulmonary blood flow was negatively correlated with the age at the time of the BCPC (r = -0.47, p = 0.02). For group A, Qp/Qs and collateral flow were both correlated with a higher oxygen saturation (r = 0.59, p = 0.02 and r = 0.49, p = 0.05, respectively). In group B, Qp/Qs correlated with the age at the time of the Fontan completion (r = 0.81, p = 0.01).

## Conclusion

APC blood flow can be quantitatively measured non-invasively, using MRI, with sufficient accuracy. The goal of volume unloading the single ventricle by creating a cavopulmonary anastomosis is counteracted by APC flow. Progression to the BCPC at a younger age is leads to a greater degree of aortopulmonary collateral artery development.

